# Colonization of patients, healthcare workers, and the environment with healthcare-associated *Staphylococcus epidermidis* genotypes in an intensive care unit: a prospective observational cohort study

**DOI:** 10.1186/s12879-016-2094-x

**Published:** 2016-12-09

**Authors:** Micael Widerström, Johan Wiström, Helén Edebro, Elisabeth Marklund, Mattias Backman, Per Lindqvist, Tor Monsen

**Affiliations:** 1 Department of Clinical Microbiology, Unit of Research, Education and Development - Östersund, Umeå University, SE-901 85 Umeå, Sweden; 2 Department of Clinical Microbiology, Infectious Diseases, Umeå University, Umeå, Sweden; 3 Department of Clinical Microbiology, Umeå University, Umeå, Sweden; 4 Department of Clinical Microbiology, Unit of Research, Education and Development-Östersund, Umeå University, Umeå, Sweden; 5 Department of Anesthesiology and Intensive Care, Unit of Research, Education and Development-Östersund, Umeå University, Umeå, Sweden

**Keywords:** *Staphylococcus epidermidis*, Cross infection/epidemiology, Cross infection/infection & control, Pulsed-field gel electrophoresis (PFGE), Molecular epidemiology, Multilocus sequence typing (MLST), Healthcare-associated infections, Infectious Disease Transmission, Professional-to-Patient, Intensive Care Units, Environmental Microbiology

## Abstract

**Background:**

During the last decades, healthcare-associated genotypes of methicillin-resistant *Staphylococcus epidermidis* (HA-MRSE) have been established as important opportunistic pathogens. However, data on potential reservoirs on HA-MRSE is limited. The aim of the present study was to investigate the dynamics and to which extent HA-MRSE genotypes colonize patients, healthcare workers (HCWs) and the environment in an intensive care unit (ICU).

**Methods:**

Over 12 months in 2006–2007, swab samples were obtained from patients admitted directly from the community to the ICU and patients transferred from a referral hospital, as well as from HCWs, and the ICU environment. Patients were sampled every third day during hospitalization. Antibiotic susceptibility testing was performed according to EUCAST guidelines. Pulsed-field gel electrophoresis and multilocus sequence typing were used to determine the genetic relatedness of a subset of MRSE isolates.

**Results:**

We identified 620 MRSE isolates from 570 cultures obtained from 37 HCWs, 14 patients, and 14 environmental surfaces in the ICU. HA-MRSE genotypes were identified at admission in only one of the nine patients admitted directly from the community, of which the majority subsequently were colonized by HA-MRSE genotypes within 3 days during hospitalization. Almost all (89%) of HCWs were nasal carriers of HA-MRSE genotypes. Similarly, a significant proportion of patients transferred from the referral hospital and fomites in the ICU were widely colonized with HA-MRSE genotypes.

**Conclusions:**

Patients transferred from a referral hospital, HCWs, and the hospital environment serve as important reservoirs for HA-MRSE. These observations highlight the need for implementation of effective infection prevention and control measures aiming at reducing HA-MRSE transmission in the healthcare setting.

**Electronic supplementary material:**

The online version of this article (doi:10.1186/s12879-016-2094-x) contains supplementary material, which is available to authorized users.

## Background

In humans, *Staphylococcus epidermidis* is a ubiquitous commensal of the skin and mucous membranes, but also an important pathogen causing a variety of healthcare-associated infections [1]. Epidemic clonal lineages of methicillin-resistant *S. epidermidis* (MRSE) have been identified in different parts of the world that seem confined to healthcare settings [2–7]. The reservoir of these healthcare-associated MRSE (HA-MRSE) clones is unknown. It has been speculated that they evolved and disseminated in the hospital setting through a process involving adaptation and selection [7, 8]. Previous studies have shown that antibiotic treatment and hospitalization rapidly affect the patient *S. epidermidis* microbiota [9, 10]. Similarly, the presence of MRSE nasal carriage is clearly higher among HCWs (30–94%) compared with non-HCWs (19–40%) [2, 11–13]. In addition there is data to suggest that HCWs acts as a reservoir and vector for the transmission of pathogenic *S. epidermidis* genotypes [14]. However, there are also studies that have failed to demonstrate convincing relationship between genotypes of S*. epidermidis* causing clinical infections in patients and genotypes identified among HCWs [15].

Nevertheless, there is still limited data utilizing more modern molecular epidemiological methods characterizing the dynamics of *S. epidermidis* colonization in the healthcare setting. We hypothesized that hospitalised patients, healthcare workers (HCWs) and the hospital environment may act as reservoirs for HA-MRSE genotypes, which readily colonize patients newly admitted to hospitals.

The aim of the current study was to determine the prevalence of HA-MRSE genotypes during the first 2 weeks of hospitalization in patients admitted to an intensive care unit (ICU) directly from the community compared to patients transferred from a referral hospital, HCWs and the environment in an ICU setting.

## Methods

### Setting

Östersund Hospital (ÖH) is a 400-bed secondary hospital that includes an eight-bed ICU providing critical care services to residents of Jämtland County, Sweden (population 127,000). The referral University Hospital of Umeå (UH) is located approximately 350 km to the northeast. The study was conducted between July 1, 2006 and June 30, 2007.

### Patients

Two categories of patients were eligible for the study: (i) those admitted to the ICU ≤24 h immediately preceding hospitalization at ÖH, henceforth called community patients, and (ii) those transferred to the ICU from the referral hospital UH, called referral patients. Consecutive patients ≥18 years of age with expected length of ICU stay of ≥7 days were asked to participate in the study and were given verbal and written information before enrolment. Written informed consent to participate was obtained from the patients themselves or was provided by the guardians of the patients who were unable to respond on their own behalf. Gender, age, and on-going antibiotic treatment were recorded. Medical records were reviewed regarding antibiotic treatment and/or hospitalization during the preceding 12 months.

On days 1, 3, 5, 8, 11, and 14 during the ICU stay samples for culture were obtained from each patient from the following sites: nostril, back of one hand, axilla, the perineum, and, when applicable, at the insertion site of a peripheral, a central venous and an arterial catheter, from urine and from the endotracheal tube. The study was approved by the Research Ethics Committee of the Faculty of Medicine, Umeå University, Umeå, Sweden (No. 07–089 M).

### Health care workers

A majority of the HCWs at the ICU (37/61) agreed to participate in the study: three of 16 medical doctors (MDs) (19%), 23 of 30 nurses (77%), and 11 of 15 assistant nurses (73%). Participation was voluntary, anonymous, and only gender, profession, and years of employment at the ICU were recorded. Swabs were collected from the nostrils and the back of one hand of each HCW, preferably at the start of a work shift. The ICU study nurse or a colleague obtained these samples, during three periods: July 2006, December 2006, and June 2007. Nasal carriage patterns were defined as follows: “persistent carriage” = isolation of the same genotype of *S. epidermidis* in ≥ two of the culture periods; “transient carriage” = isolation of a specific genotype of *S. epidermidis* in ≤ one of the culture periods [16].

### Environment

Fourteen environmental samples were collected at the ICU by the principal investigator on one occasion in January 2007. The samples were obtained from four telephone handsets, six computer keyboards, two ventilator panels and two infuser panels. One of the ventilators and infuser panels were located in a cleaned and vacant ICU patient room.

### Sample collection

To collect a sample, a sterile cotton swab soaked in 0.9% sterile sodium chloride solution was rubbed over an area of 1–2 cm^2^, placed in transport medium (Copan, Brescia, Italy) and delivered to the laboratory within 1 h. Each sample was plated using triple streak technique on a separate plate of Iso-Sensitest agar (Oxide Ltd, Basingstoke, UK). A 10-μg cefoxitin disc was placed at the periphery of the primary streak on the agar, and the plate was incubated overnight in ambient air at 35 °C. Based on morphology, four colonies with the macroscopic appearance of coagulase-negative staphylococci (CoNS) situated as close as possible to the cefoxitin disc on each plate were randomly selected for further investigation. Samples where MRSE isolates were not detected were further examined using selective enrichment broth. A 10 ul loop of bacteria from the primary streak were suspended in 5 ml PBS (0.5 McFarland standard) of which 100 μl was added into in a selective enrichment broth (brain heart infusion and 4 mg/ ml cefoxitin) and incubated for 24–48 h in air at 35 °C. Then, 100 μl of the broth was inoculated onto Iso-Sensitest agar with a 10-μg cefoxitin disc and re-examined for presence of MRSE.

### Identification and antibiotic susceptibility testing of S. epidermidis strains

CoNS were identified by standard methods (colony morphology, catalase positive, DNase negative) [17], and further identified to species level by matrix-assisted laser desorption/ionization time-of-flight mass spectrometry (MALDI-TOF MS) and the Biotyper 2.0 database (Bruker Daltronics, Bremen, Germany) [18]. A score of ≥2 was accepted for identification. All isolates were tested for antimicrobial susceptibility to cefoxitin, clindamycin, co-trimoxazole, gentamicin, and fusidic acid according to the guidelines of the European Committee on Antimicrobial Susceptibility Testing (EUCAST) (v 5.0, www.eucast.org). Constitutive and inducible resistance to clindamycin was determined with the D-shaped disc diffusion method (Oxoid AB, Sweden). After initial identification, isolates were stored at −80 ° C pending further analysis. Multidrug-resistance (MDR) were defined as resistance to cefoxitin and ≥3 other classes of antimicrobial agents. When estimating the MRSE prevalence and the prevalence of resistance to other antimicrobials among patients per sampling day, the *S. epidermidis* isolate exhibiting resistance to highest number of antimicrobials was used.

### Pulsed-field gel electrophoresis and multilocus sequence typing

PFGE and MLST were performed as previously described [19]. All environmental MRSE isolates (*n* = 25), MRSE isolates that exhibited disparate susceptibility patterns from each plate obtained from the HCW (*n* = 132), community patients (*n* = 123), and referral patients on day 1 (*n* = 22) were characterized using pulsed-field gel electrophoresis (PFGE). PFGE types that included at least three MRSE isolates were analysed by multilocus sequence typing (MLST). Sequence types (STs) were assigned using the *S. epidermidis* MLST database (http://www.mlst.net). Clonal complexes (CC) were determined using the eBURST algorithm. HA-MRSE isolates were defined as belonging to clonal complex 2 (CC2) [6].

### Statistical analysis

All statistical analyses were conducted using the SPSS software package (version 20.0; SPSS, Chicago, IL, USA). Fisher’s exact test was applied to assess associations in all two-way tables. A *p*-value of <0.05 was considered significant.

## Results

The community patients comprised nine consecutive patients (eight women, one male) with a mean age of 70 years (range 55–84 years) and median length of ICU stay of 5 days (range 1–14 days). The referral-group included five patients (four men, one woman) with a median age of 67 years (range 22–73 years) and a median stay of 9 days (range 1–10 days) at UH before transfer (Additional file 1). Mortality among the included patients during ICU stay was low, only case 2 died (day 2).

In total, 570 cultures were obtained during the study, among which CoNS were identified in 362. From these samples 1167 CoNS isolates were obtained, 934 (80%) were identified as *S. epidermidis*, of which 620 (66%) were methicillin-resistant (Table 1).

**Table 1:** Distribution of cultures, coagulase-negative staphylococci (CoNS), *Staphylococcus epidermidis* and methicillin-resistant *S. epidermidis* (MRSE) according to source

Source (n)	Cultures *n*	CoNS *n*	*S. epidermidis n* (%)	MRSE *n* (%)
Medical Doctor (3)	18	43	43 (100)	13 (30)
Nurse (23)	94	249	234 (94)	166 (71)
Assistant nurse (11)	47	173	154 (89)	106 (69)
Environment (14)	14	41	25 (61)	24 (96)
Referral patients (5)	151	222	153 (69)	140 (92)
Community patients (9)	246	439	325 (74)	171 (53)
Total	570	1167	934 (80)	620 (66)

CoNS coagulase-negative staphylococci, MRSE methicillin-resistant *Staphylococcus epidermidis*

**Fig. 1:**
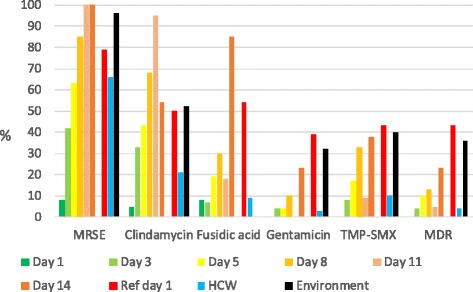
Proportion of *S. epidermidis* isolates exhibiting antimicrobial resistance according to source

The MRSE prevalence among community patients was 22% at day 1 of hospitalization, 86% at day 3 and 100% at day 5 and onwards (Table 2). MRSE prevalence in referral patients, HCW and the environment were 60, 92 and 50%, respectively. The phenotypic antibiotic resistance profile among all MRSE isolates in respective group is depicted in Fig. 1. MDR *S. epidermidis* was significantly more common in referral patients day 1 and in the environment compared with those obtained from community patients and HCWs (*p* < 0.0001) (Fig. 1). Genotyping of 238 MRSE isolates demonstrated that five STs comprised 64% (152/238) of the isolates: ST5 (*n* = 63; 26%), ST215 (*n* = 28; 12%), ST2 (*n* = 25; 11%), ST38 (*n* = 19; 8%), and ST22 (*n* = 17; 7%). The HA-MRSE prevalence among community patients was 11% (1 of 9) at day 1 of hospitalization, 86% (6 of 7) at day 3, 83% at day 5 and 100% at day 8 compared with 40% (2 of 5) in referral patients, 92% in HCW and the 43% in samples from the ICU environment (Table 2).

**Table 2:** Prevalence of antimicrobial resistance and healthcare-associated *S. epidermidis* ST types according to according to source

Source (n)	Antimicrobial resistance^a^ %					Healthcare-associated
	methicillin	clindamycin	fusidic acid	gentamicin	TMP-SMX	ST types^b^ %
Community patients						
Day 1 (9)	22	11	11	0	0	11
Day 3 (7)	86	86	57	29	57	86
Day 5 (6)	100	83	50	33	67	83
Day 8 (2)	100	100	100	100	100	100
Day 11 (2)	100	100	100	0	100	50
Day 14 (2)	100	100	100	50	50	50
Referral patients day 1 (*n* = 5)	60	60	60	60	60	40
Environment (11)	50	29	0	14	29	43
Medical doctor (3)	100	67	0	0	33	67
Nurse (23)	91	57	39	26	39	100
Assistant nurse (11)	100	36	45	22	36	91

^a^When estimating the prevalence of antimicrobial resistance according to source, the *S. epidermidis* isolate exhibiting resistance to highest number of antimicrobials was used

^b^defined as belonging to clonal complex 2 (CC2)

### Patients

*S. epidermidis* from referral patients on day 1 of admission to the ICU showed significantly more often resistance to all tested antimicrobial agents compared with *S. epidermidis* from community patients (*p* < 0.0001) (Fig. 1). At day 3 of admission no significant difference in methicillin-resistance was identified when comparing the two groups of patients (Table 2). At day 5 of admission *S. epidermidis* from community patients still showed significant lower frequency of resistance to fusidic acid (*p* = 0.0012), gentamicin (<0.0001) and co-trimoxazole (*p* = 0.0092), but at day 8 only the difference in gentamicin resistance was detected (*p* = 0.0067). From day 11 post admission and onwards no difference in antimicrobial resistance was identified comparing the community patients and referral group at day 1. However, MDR was still more frequent among referral group at day 1 compared with community patients at day 11 (*p* = 0.0028) (Fig. 1).

Among the referral patients, HA-MRSE ST215 was identified in referral case 1 in a wound and at the insertion site of the central venous catheter. Referral case 4 was colonized with HA-MRSE ST215 in the perineum, the axilla and the insertion site of the central venous catheter; also ST2 were obtained from the hand and axilla samples (Table 3). No HA-MRSE was identified in the three remaining referral patients. However, MDR *S. capitis* were identified in samples from hand and axilla in case 12 and MDR *Staphylococcus heamolyticus* (hand, nose and axilla) in referral case 7 (data not shown). No MRSE or other MR-CoNS were identified in referral case 8, whom only had a length of stay at the referral hospital of <1day. In comparison, HA-MRSE was identified at day 1 in only 1 of 9 community patients (case 11, ST5, hand sample). At day 3 HA-MRSE were identified in 6 of 7 included community cases (Table 3). MR *Staphylococcus similans* but no MRSE were identified in case 10 (Additional file 1). The colonizing ST types were frequently identified in the community cases at the same sample site at day 5, 8, 11 and 14 (when applicable) and additional ST types emerged in sampling sites in four of these case (case 3, 5, 6 and 9) (Table 3).

**Table 3:** Distribution of MRSE ST types according to source of culture

MRSE	ICU Cases							ICU environment
	Community						Referral	
ST type	Day 1	Day 3	Day 5	Day 8	Day 11	Day 14	Day 1	
5	C11:H	C5: N, PVC	C5: N, H, PVC, AC,	C5: A, P, PVC,	C5: CVC, T,			Respiratory panel,
			P, A,W	W, T	H, N			C-unit (empty)
			C6: A					
			C9: N, H	C9: H, W	C9: T, W	C9: T		
2		C3: W, T,	C3: W, U				C4: H, A	Infusor panel,
		1CVC						B-unit
		C6: N	C6: N, P					Respiratory panel,
								B-unit
								Telephone, A-unit
215			C5: P, CVC	C5: CVC		C5: P,	C4: A, P,	Keyboard, A-unit
						PVC	CVC	
							C1: CVC, W	
17		C2: CVC	C5: A, AC, H, W	C5: N, W	C5: N	C5: N, H		
		C9: H	C9: AC, H, W	C9: W		C9: N		
		C13: N, P	C13: H					
22			C3: N					
			C5: N					
81		C13: H, N, T	C13: N					

*MRSE* Methicillin resistant *S. epidermidis*, *ST* Sequence type, *A* Axilla; AC, arterial catheter, *CVC* Central venous catheter, *H* Hand, *PVC* Peripheral venous catheter, *N* Nose, *P* Perineum, *T* Trachea, *U* Urine, *W* Wound

### Health care workers

**Fig. 2:**
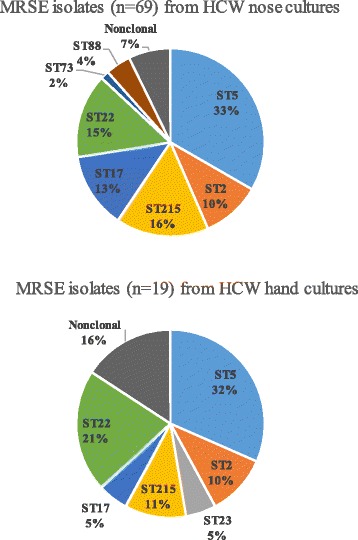
Distribution of sequence types (STs) among methicillin-resistant *S. epidermidis* (MRSE) identified in a subset of 111 cultures from the nose and hands of 37 ICU health care workers (HCW) at Östersund Hospital

Thirty of the 37 HCWs were sampled three times during the study period. Of the remaining seven, five nurses and one assistant nurse were each sampled twice, and one nurse was sampled once. Almost all (33/37, 89%) of the HCWs were nasal carriers of ≥1 HA-MRSE genotype: ST5 were identified in 14 (38%), ST215 in 9 (24%), ST22 in 8 (22%), ST2 in 6 (16%), ST17 in 5 (14%), ST218 in 2 (5%) and ST23, ST73, ST88 in one HCW, respectively. Persistent nasal carriage of HA-MRSE genotypes were identified in 19/36 (53%) HCW that were sampled >1 occasion: ST5 (*n* = 8), ST22 (*n* = 3), ST17 (*n* = 3), ST215 (*n* = 2), ST2 (*n* = 1), ST88 (*n* = 1), and ST218 (*n* = 1). In each of 16 HCWs, nasally colonized with a specific HA-MRSE genotype, identical genotype were identified, albeit not repeatedly, in cultures from their hands (Fig. 2).

### Environment

Two of 14 environment samples were negative (two keyboards in the ICU control room) and no CoNS were identified in cultures from the keyboard in the vacant ICU room. The eleven remaining investigated objects yielded 41 CoNS isolates, a majority of which (38/41, 93%) were MR-CoNS; 19/41 (46%) were MDR. HA-MRSE genotypes (Table 2) and other MDR-CoNS (*S. heamolyticus, Staphylococcus hominis* and *S. similans)* were isolated from several frequently touched fomites in the ICU.

## Discussion

We have previously demonstrated the occurrence, persistence, and potential dissemination of HA-MRSE genotypes within hospitals in northern Europe and Australia [19–21]. The present study show that the prevalence of MRSE carriage was low in patients newly admitted to the ICU from the community, but the majority was subsequently colonized with HA-MRSE genotypes within 3 days of hospitalization. In addition, the fomites in the ICU, the patients transferred from the referral hospital, and the ICU HCWs were frequently colonized with HA-MRSE genotypes. These data indicate that there is a need to develop and implement infection control measures preventing cross-transmission of HA-MRSE genotypes in the healthcare setting [22]. This is of particular importance since genotypes in CC2 lineage comprise the majority of HA-MRSE infections, often display MDR phenotype and are common among emerging linezolid-resistant MRSE isolates [2, 6, 8, 23, 24].

Even short treatment with antibiotics may affect *S. epidermidis* microbiota [9, 10]. All patients in the current study were treated or had recently been treated with antibiotics, which may have influenced the studied MRSE colonization dynamics. But even use of enrichment broth techniques did not enable us to detect the presence of MRSE at admission to the ICU in seven of the nine community patients, implying that six of these seven subjects were subsequently colonized with HA-MRSE within 3 days of hospitalization by cross-contamination from the hospital environment or HCWs. Corroborating our results, prolonged hospitalization have been correlated with both the emergence of HA-MRSE and decreased clonal diversity [25, 26]. Minimizing length of stay prior to surgery may be one factor to consider in reducing colonization of HA-MRSE in patients. At admission, two of five referral patients were colonized with HA-MRSE and two other with MDR *S. capitis* and *S. heamolyticus* respectively, which also have been recognized causing HA infections and outbreaks [27–29]. These data support the hypothesis that the transfer of patients between hospitals may have an important contribution to the dissemination of HA-MRSE genotypes [21, 30]. Furthermore, all patients continued to be colonized with these HA-MRSE genotypes for the remaining length of stay even though additional HA-MRSE genotypes emerged in individual patients. Interesting, we were not able to identify MRSE in two of the referral cases, which were colonized with other MDR CoNS. Why individual patients become and stay colonized with specific HA-MRSE genotypes or other MDR CoNS species remains to be investigated.

It has previously been documented that, compared to non-healthcare professionals, HCWs have higher prevalence of both nasal MRSE colonization and carriage of HA-MRSE genotypes that are prevalent among the patients that they care for [2]. These genotypes are relatively quickly established in newly graduated HCWs and are re-established in HCWs returning to work after a vacation [14, 31, 32]. This observation corroborate our results showing that a large proportion of HCWs were persistent nasal carriers of HA-MRSE genotypes, and that the genotypes identified in nasal cultures frequently were identical to the genotypes found on the individual HCW’s hand. These findings further substantiate the assumption that HCWs may act as an important reservoir and cause of cross-transmission of HA-MRSE genotypes in the healthcare setting. All HCWs in the present study had been employed at the ICU for more than 10 years, which may have contributed to the high prevalence of HA-MRSE carriage.

HA-MRSE belonging to ST2, ST5, or ST215 was isolated from several fomites in the ICU. There is increasing evidence that environmental colonization may play a significant role in the transmission of MDR bacteria such as MRSA and VRE [33, 34], whereas data is limited regarding the contribution of environmental colonization to the spread of *S. epidermidis* [35–37]. Studies have shown that *Staphylococcus* spp. constitute an important part of the microbiota colonizing the hospital environment [38]. This is apparent even after routine daily cleaning or after adding copper to surfaces that are frequently touched in hospital settings [39, 40]. However, the cited reports do not include information about species identification or molecular epidemiology. Further studies are needed to evaluate the contribution of environmental colonization to the dissemination of HA-MRSE genotypes.

The present investigation has several potential limitations. First, MRSE were used exclusively as a marker of HA-strains and thereby excluded the possibility of detecting genetically closely related methicillin-susceptible *S. epidermidis*. Secondly, this was a single-centre study performed at a county hospital level including a limited number of patients, some hospitalized for only 1 or 2 days. Lastly, to address our hypothesis, we genotyped only a subset of the identified MRSE isolates. Hence, we did not obtain a complete overview of the molecular epidemiology of these strains. All of these factors may limit the generalizability of the current results. However, although a limited number of patients were evaluated, the number of isolates included for the majority of patients was substantial which provided more detailed information of the molecular epidemiology in each studied patient. New less cumbersome genotyping techniques would indeed facilitate future surveys aiming at a more complete picture of the *S. epidermidis* colonization dynamics in the healthcare setting.

## Conclusion

In conclusion, our findings suggest that patients referred from other hospitals, HCWs and the hospital environment serve as important reservoirs for HA-MRSE. These genotypes colonized the majority of newly admitted patients within 3 days of hospitalisation. Further studies are needed to confirm the present results, which may have implications for infection control measures aiming at reducing HA-MRSE transmission in the healthcare setting.

## Additional file

Additional file 1: Epidemiological, clinical and microbial data for the 14 consecutive patients included in the study. (DOCX 32 kb)

## Abbreviations

CoNS: Coagulase-negative staphylococci
HA-MRSE: Healthcare-associated methicillin-resistant *Staphylococcus epidermidis*
HCWs: Healthcare workers
ICUs: Intensive Care Units
MDR: Multidrug-resistance
MLST: Multilocus Sequence Typing
MR: Methicillin-resistant
MRSE: Methicillin-resistant *Staphylococcus epidermidis*
PFGE: Pulsed-field gel electrophoresis
ST: Sequence Type
