# Supplementary material for: Colonization of patients, healthcare workers, and the environment with healthcare-associated Staphylococcus epidermidis genotypes in an intensive care unit: a prospective observational cohort study
Source: BMC Infect Dis. 2016 Dec 9;16:743. doi: 10.1186/s12879-016-2094-x (PMC5148920; doi:10.1186/s12879-016-2094-x)
Supplement: Additional file 1: — Epidemiological, clinical and microbial data for the 14 consecutive patients included in the study. (DOCX 32 kb) [file 12879_2016_2094_MOESM1_ESM.docx]

Additional file 1. Epidemiological, clinical and microbial data for the 14 consecutive patients included in the study

| Case no. | Antibiotic treatment | Hospitalization | Admission LOS^a^ (days UH^b^ / ICU ÖH^c^) | No. of samples/no. of samples with identified CoNS^d^/no. of CoNS isolates methicillin resistant (%) | *S. epidermidis* isolated on day 1 of hospitalization | *S. epidermidis* isolated on day 3 of hospitalization |  | Antibiotic treatment^e^ | |
| --- | --- | --- | --- | --- | --- | --- | --- | --- | --- |
|  |  |  |  |  |  |  |  | at UH (no. of days) | at ÖH ICU (no. of days) |
|  | during previous 12 months (no. of days) | |  |  |  |  |  |  |  |
|  |  |  |  |  |  |  |  |  |  |
| 1 | No | No | 10/8 | 30/8/28 (79) | MRSE^f^ ST 215 | Referral patient, no CoNS isolates evaluated using PFGE and MLST^h^ |  | CXM GEN (10) | None |
|  |  |  |  |  |  |  |  |  |  |
| 2 | Yes (FCX^e^), 5 months ago (7) | Yes | 0/3 | 14/9/36 (31) | No MR-CoNS^g^ | MRSE ST17 MR-*S. capitis* |  | NA^i^ | CXM MNZ (3) |
|  |  |  |  |  |  |  |  |  |  |
| 3 | No | No | 0/5 | 28/18/73 (18) | No MR-CoNS | MRSE ST2 MR-*S. haemolyticus* |  | NA | CXM (5) |
|  |  |  |  |  |  |  |  |  |  |
| 4 | No | Yes, 2 months ago (15) | 9/14 | 41/36/111 (94) | MRSE ST 2 and ST215 | Referral patient, no CoNS isolates evaluated using PFGE and MLST |  | MEM (9) | MEM VAN MNZ (10)  CLI LVX (4) |
|  |  |  |  |  |  |  |  |  |  |
| 5 | No | Yes, 3 months ago (7) | 0/14 | 47/33/118 (85) | MRSE non clonal | MRSE ST5 |  | NA | CXM (3) CMX LVX (2) PEN (9) |
|  |  |  |  |  |  |  |  |  |  |
| 6 | No | No | 0/5 | 26/9/34 (56) | No MR-CoNS | MRSE ST2 |  | NA | CMX LVX (2) PEN (2) |
|  |  |  |  |  |  |  |  |  |  |
| 7 | Yes (NIT^e^), 8 months ago (5) | Yes, 4 months ago (12) | 8/7 | 35/30/36 (100) | MR-*S. haemolyticus* | Referral patient, no CoNS isolates evaluated using PFGE and MLST |  | TZP LVX (8) | TZP (8) |
|  |  |  |  |  |  |  |  |  |  |
| 8 | No | No | 1/3 | 7/9/33 (6) | No MR-CoNS | Referral patient, no CoNS isolates evaluated using PFGE and MLST |  | DCX MNZ (1) | DCX MNZ (3) |
|  |  |  |  |  |  |  |  |  |  |
| 9 | No | No | 0/14 | 64/29/69 (52) | No MR-CoNS | MRSE ST17 MR-*S. cohnii* MR-*S. warneri* |  | NA | CXM MNZ (5) |
|  |  |  |  |  |  |  |  |  |  |
| 10 | No | No | 0/5 | 28/8/14 (43) | MR-*S. similans* | MR-*S. similans* MR-*S. hominis* |  | NA | CXM (1) |
|  |  |  |  |  |  |  |  |  |  |
| 11 | Yes, (DOX^e^) 12 monts ago (9) | No | 0/2 | 8/4/13 (8) | MRSE ST5 | Discharged |  | NA | None |
|  |  |  |  |  |  |  |  |  |  |
| 12 | No | No | 10/5 | 28/8/14 (58) | MRSE nonclonal MR *S. capitis* | Referral patient, no CoNS isolates evaluated using PFGE and MLST |  | TZP (2) | TZP (3) |
|  |  |  |  |  |  |  |  |  |  |
| 13 | No | No | 0/5 | 25/11/45 (29) | No MR-CoNS | MRSE ST17, ST 81 |  | NA | CXM (3) |
|  |  |  |  |  |  |  |  |  |  |
| 14 | No | No | 0/1 | 4/4/10 (0) | No MR-CoNS | Discharged |  | NA | MEM (1) |

^a^LOS, length of stay

^b^UH, University Hospital of Umeå

^c^ICU, Intensive care unit; ÖH, Östersund County Hospital

^d^CoNS, coagulase-negative staphylococci

^e^Antibiotics given: FCX, flucloxacillin; CXM, cefuroxime; CLI, clindamycin; DCX, dicloxacillin; DOX, doxycycline; GEN, gentamicin; LVX, levoﬂoxacin; MEM, meropenem; NIT, nitrofurantoin; PEN, penicillin; TZP, piperacillin-tazobactam; VAN, vancomycin.

^f^MRSE, methicillin-resistant *Staphylococcus epidermidis*

^g^MS-CoNS, methicillin-sensitive coagulase-negative staphylococci

^h^PFGE, pulsed-field gel electrophoresis; MLST, multilocus sequence typing

^j^NA, not applicable
